# Is There a Nonlinear Relationship between Serum Uric Acid and Lipids in a Hypertensive Population with eGFR ≥30 ml/min/1.73 m^2^? Findings from the China Hypertension Registry Study

**DOI:** 10.1155/2020/9725979

**Published:** 2020-09-18

**Authors:** Yu Yu, Xiao Huang, Minghui Li, Congcong Ding, Lihua Hu, Xiao Zhong, Wei Zhou, Tao Wang, Lingjuan Zhu, Huihui Bao, Xiaoshu Cheng

**Affiliations:** ^1^Department of Cardiovascular Medicine, The Second Affiliated Hospital of Nanchang University, Nanchang, Jiangxi, China; ^2^Center for Prevention and Treatment of Cardiovascular Diseases, The Second Affiliated Hospital of Nanchang University, Nanchang, Jiangxi, China

## Abstract

**Background:**

Evidence regarding the nonlinear relationship between serum uric acid (SUA) and blood lipids in Chinese population with hypertension is limited. Therefore, the present study aimed to investigate whether there is a nonlinear association between SUA and lipids in Chinese hypertensive population with an estimated glomerular filtration rate (eGFR) ≥30 ml/min/1.73 m^2^.

**Methods:**

A total of 13,355 hypertensive participants with eGFR ≥30 ml/min/1.73 m^2^ were selected from the Chinese Hypertension Registry Study. Multivariate linear regression was used to examine the linear relationship between SUA and lipids. Smooth curve fitting (penalized spline method) and threshold saturation effects were used to analyze the nonlinear association between SUA and lipids.

**Results:**

In the fully adjusted model, the results showed a positive correlation between SUA and TG (*β* = 0.15; 95% CI: 0.14, 0.16) and LDL-C (*β* = 0.06; 95% CI: 0.05, 0.07), respectively. However, the relationship between SUA and HDL-C was nonlinear. The inflection point of SUA was 7.24 mg/dL. On the left side of the inflection point (<7.24 mg/dL), SUA was negatively associated with HDL-C (*β* = −0.02; 95% CI −0.02, −0.01). On the right side of the inflection point (≥7.24 mg/dL), SUA was not related to HDL-C (*β* = 0.01; 95% CI −0.01, 0.02).

**Conclusion:**

After adjusting for all covariates, SUA was positively associated with TG and LDL-C. The relationship between SUA and HDL-C was nonlinear. The negative correlation between SUA and HDL-C only existed when the SUA was less than 7.24 mg/dL in a hypertensive population with eGFR ≥30 ml/min/1.73 m^2^.

## 1. Introduction

Coronary heart disease (CHD) is the leading cause of death in the global population, of which dyslipidemia was reported as the most important risk factor for CHD [1, 2]. Besides, serum uric acid (SUA) is the end product of purine metabolism, and elevated levels of SUA are generally considered as risk factors for CHD [3, 4]. Blood lipids and SUA are closely related, but this relationship has been controversial. Several recent studies showed that elevated SUA was positively associated with triglycerides (TG) and high-density lipoprotein cholesterol (HDL-C) [5–7]. Other studies reported that SUA was negatively associated with HDL-C, while SUA was not related with TG and low-density lipoprotein cholesterol (LDL-C) [8, 9]. In addition, some studies reported a nonlinear relationship between SUA level and the occurrence of CHD events [10, 11], in which blood lipids are the closest risk factor for CHD [12], and it is unclear whether there also is a nonlinear relationship between SUA and blood lipids.

Epidemiological survey showed that 23.2% of Chinese adults had hypertension, which caused a great social burden [13]. It should be noted that patients with hypertension often have dyslipidemia and hyperuricemia [14, 15]. However, to our knowledge, few studies examined the relationship between SUA and blood lipids in patients with hypertension. In addition, SUA cannot be effectively excreted through the kidney when the estimated glomerular filtration rate (eGFR) is <30 ml/min/1.73 m^2^, resulting in abnormal elevation of SUA levels [16]. In the present study, we tried to examine the relationship between SUA and blood lipids (including TG, LDL-C, and HDL-C) in a hypertensive population with eGFR ≥30 ml/min/1.73 m^2^.

## 2. Materials and Methods

### 2.1. Study Design and Participants

The study data were drawn from the China Hypertension Registry study. The primary objective of this study was to establish a Chinese population-based cohort of hypertensive patients to collect baseline data and investigate the current situation of hypertensive patients in China. A total of 14,268 study participants were recruited from March 2018 to August 2018 in Wuyuan, Jiangxi Province, China. Details of this study are shown in detail in the previously published literature [17].

The study protocol was approved by the Ethics Committee of the Anhui Medical University Biomedical Institute. All study participants completed written informed consent.

### 2.2. Laboratory Assay

Venous blood samples were collected from all study participants in fasting by trained study staff. Blood lipids, SUA, fasting blood glucose (FBG), homocysteine (Hcy), liver function, and kidney function were measured with an automatic clinical analyzer (Beckman Coulter, USA) in Biaojia Biotechnology Laboratory, Shenzhen, China.

### 2.3. Other Variables

Body mass index (BMI, kg/m^2^) was calculated by dividing weight by the square of height. Blood pressure (BP, mmHg) was measured using an electronic sphygmomanometer. Information on age, sex, smoking, alcohol use, history of disease, and history of taking medicine was obtained through standard questionnaires.

### 2.4. Statistical Analysis

In order to reduce the impact of the maximum and minimum values on the statistical results, we removed the 2.5% interval on both sides of the SUA value. The baseline characteristics of the study participants were described according to the tertiles of SUA values. To examine the correlation between SUA and lipids, our statistical analyses consisted of 3 steps. Step 1: Multivariate linear regression was used to analyze the linear relationship between uric acid and blood lipids (including TG, LDL-C, and HDL-C). Crude model (unadjusted), Model 1 (minor adjusted), and Model 2 (fully adjusted) were constructed to explore the relationship between SUA and lipids under the adjustment of different variables. The *P* values for trends were used to determine whether there was a dose-dependent relationship between SUA and lipids. Step 2: Smooth curve fitting (penalized spline method) was used to address for nonlinear association between SUA and lipids (including TG, LDL-C and HDL-C). If nonlinearity was detected, the threshold effect analysis was specifically used to accurately describe nonlinear relationships. The inflection point is calculated by recursive algorithm, and then, a two-piecewise Cox proportional hazards model was constructed on both sides of the inflection point. The threshold level was determined by choosing the inflection point which provided the maximum model likelihood, using the trial and error method, along with a log likelihood ratio test comparing the one-line Cox proportional hazards model with a two-piecewise Cox proportional hazards model to examine the statistical significance [18]. Step 3: Subgroup analysis was used to assess whether the relationship was stable in different variable stratifications.

All statistical analyses were performed using the statistical package R (http://www.R-project.org, The R Foundation) and EmpowerStats (http://www.empowerstats.com, X&Y Solutions, Inc., Boston, MA). Statistical significance was defined as two-tailed *P* < 0.05.

## 3. Results

### 3.1. Baseline Characteristics of Participants

Baseline characteristics of selected participants according to tertiles of SUA are shown in Table 1. The average age of the 13,355 selected participants was 63.77 ± 9.33 years, and about 47.18% of them were male. The mean ± SD values for SUA, TG, LDL-C, and HDL-C was 6.97 ± 1.74 mg/dL, 1.80 ± 1.25 mmol/L, 2.98 ± 0.81 mmol/L, and 1.57 ± 0.42 mmol/L, respectively. The participants with higher SUA levels had higher values for age, male, smoking, alcohol use, CHD, DM, antihypertensive drugs, BMI, mean DBP, TC, TG, LDL-C, Hcy, and FBG and lower values for mean SBP, HDL-C, and eGFR (all *P* < 0.05).

### 3.2. Relationship between SUA and Blood Lipids

Table 2 shows the results of multivariate linear regression analysis of the relationship between SUA and blood lipids. Crude model, model 1, and model 2 are unadjusted model, minor adjusted model, and fully adjusted model, respectively. When the outcome variable was TG, the association between SUA and TG was positive in model 3 (*β* = 0.15, 95% CI: 0.14, 0.16). Next, SUA was converted from continuous variable to tertile. Compared with T1 (<5.57 mg/dL), the estimated increases of TG in the T2 and T3 groups were 0.26 and 0.54, respectively (T2: *β* = 0.26, 95% CI: 0.21, 0.31; T3: *β* = 0.54, 95% CI: 0.48, 0.60; *P* for trend <0.001). Similarly, the relationship between SUA and LDL-C was positive in model 3 (*β* = 0.06, 95% CI: 0.05, 0.07). Compared with T1 (<5.57 mg/dL), the estimated increase of LDL-C in the T2 and T3 groups were 0.13 and 0.22, respectively (T2: *β* = 0.13, 95% CI: 0.10, 0.16; T3: *β* = 0.22, 95% CI: 0.18, 0.25; *P* for trend <0.001). In contrast, SUA was negatively correlated with HDL-C, but the association between SUA and HDL-C was not significant in model 3 (*β* = −0.01, 95% CI: −0.01, 0.01). Compared with T1 (<5.57 mg/dL), the estimated decreases of HDL-C in the T2 and T3 groups were 0.02 and 0.02, respectively (T2: *β* = −0.02, 95% CI: −0.04, −0.01; T3: *β* = −0.02, 95% CI: −0.04, 0.01). The *P* for trend was 0.104, indicating that there may be a nonlinear relationship between SUA and HDL-C.

### 3.3. Analyses of Nonlinear Relationship between SUA and HDL-C

**Figure 1:**
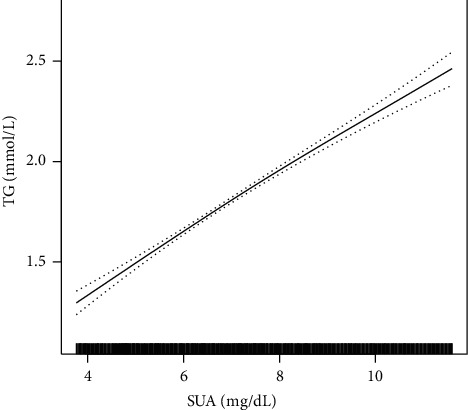
The positive association between SUA and TG in a hypertensive population. Adjusted for age, sex, smoking, alcohol use, stroke, diabetes, antihypertensive drugs, lipid-lowering drugs, glucose-lowering drugs, BMI, SBP, DBP, Hcy, FBG, and eGFR.

**Figure 2:**
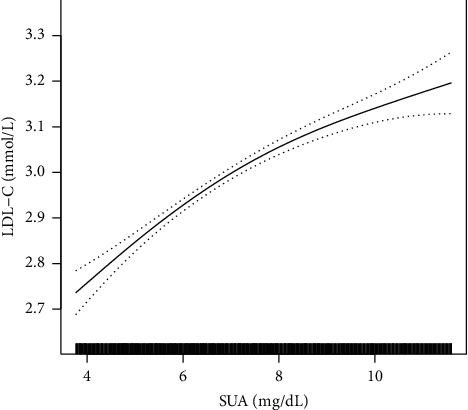
The positive association between SUA and LDL-C in a hypertensive population. Adjusted for age, sex, smoking, alcohol use, stroke, diabetes, antihypertensive drugs, lipid-lowering drugs, glucose-lowering drugs, BMI, SBP, DBP, Hcy, FBG, and eGFR.

**Figure 3:**
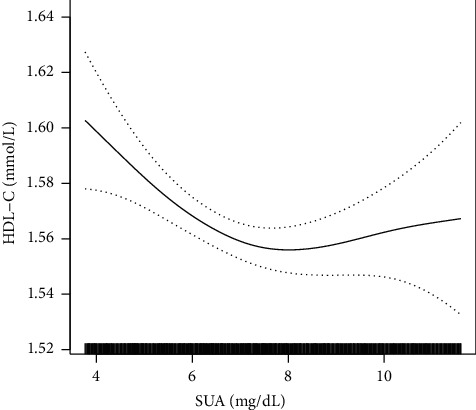
The nonlinear relationship between SUA and HDL-C in a hypertensive population. Adjusted for age, sex, smoking, alcohol use, stroke, diabetes, antihypertensive drugs, lipid-lowering drugs, glucose-lowering drugs, BMI, SBP, DBP, Hcy, FBG, and eGFR.

In the present study, the nonlinear relationships between SUA and TG, LDL-C, and HDL-C was shown by smooth curve fitting (Figures 1–3).  Figure 3 shows a nonlinear relationship between SUA and HDL-C after adjusting all covariates. Furthermore, we used a two-piecewise linear regression model to fit the nonlinear relationship between SUA and HDL-C (Table 3). The *P* value for the log likelihood ratio test is <0.05, indicating that the relationship between SUA and HDL-C was nonlinear. The inflection point of SUA was 7.24 mg/dL. On the left of the inflection point, the effect size and 95% CI were −0.02 and −0.02 to −0.01. However, on the right side of the inflection point, the association between SUA and HDL-C was not significant (*β* = −0.01, 95% CI: −0.01, 0.02).

### 3.4. Subgroup Analysis

Table S2 shows that the relationship between SUA and TG has significant differences under age, BMI, diabetes, and eGFR stratification.  Table S3 shows that the relationship between SUA and LDL-C has significant differences under BMI, diabetes, and smoking stratification.  Table S4 shows that the relationship between SUA and HDL-C has significant differences under age and eGFR stratification (*P* for interaction <0.05).

## 4. Discussion

In the present study, our results suggested a positive association between SUA and TG and LDL-C, respectively. However, there was a nonlinear relationship between SUA and HDL-C. The negative relationship between SUA and HDL-C only existed when SUA was less than 7.24 mg/dL in hypertensive population with eGFR ≥30 ml/min/1.73 m^2^. The *β* (95% CI) values were −0.02 (−0.02, −0.01) on the left side of the inflection point.

Previous studies have examined the relationship between SUA and lipids, but these studies have inconsistent conclusions. Li et al. [8] included 1,026 diabetic patients for analysis (mean age 65.57 ± 11.70 years) and found a negative correlation between SUA and HDL-C, but the relationship between SUA and TG and LDL-C was not significant. Kim et al. [9] analyzed data from 504 diabetic patients (mean age: 57.3 ± 13.9 years) and found a negative association between SUA and HDL-C, but SUA was not related to LDL-C. Tian et al. [19] included 15,401 middle-aged and elderly patients for analysis and found a positive correlation between SUA and TG but a negative correlation between SUA and HDL-C. Besides, to our knowledge, few studies have directly explored the specific relationship between SUA and lipids in Chinese hypertensive population. Hence, the exact relationship between SUA and lipids in hypertensive patients remains unclear indeed. Of note, several studies have reported a nonlinear relationship between SUA and CVD [10, 11]. Because lipids and CVD are closely related, there may also be a nonlinear relationship between SUA and lipids. To test this hypothesis, we used sufficient statistical methods to reexamine the relationship between SUA and lipids and to explore the possibility of nonlinearity.

In the present study, we found a positive correlation between SUA and TG. Consistent with our study, some previous studies have also found this relationship [5, 6, 19]. Several potential mechanisms can explain this relationship. The high levels of SUA may limit the catabolism of triacylglycerol by reducing the activity of lipoprotein lipase, thus leading to increased TG levels [20]. In addition, higher SUA levels can damage pancreatic *β* cells and reduce insulin receptor sensitivity, which can lead to reduced lipolysis and consequently increased TG levels [21]. Meanwhile, we also found a positive correlation between SUA and LDL-C. This relationship has been found in the normal population [5]. This may be because SUA as an important antioxidant in serum effectively prevented the oxidation of LDL-C, thus leading to the elevation of LDL-C level [22].

Of note, first, we found a nonlinear relationship between SUA and HDL-C in a hypertensive population. In the SUA <7.24 mg/dL group, there was a negative association between SUA and HDL-C. This relationship may be caused by adiponectin. Adiponectin can increase the level of HDL-C by accelerating the oxidation of free fatty acids [23]. However, higher SUA leads to a decrease in adiponectin levels, which in turn reduces HDL-C levels. In the SUA ≥7.24 mg/dL group, SUA was positively associated with HDL-C, but this relationship was not statistically significant. In our study, higher SUA levels were associated with alcohol use (Table S1). Appropriate alcohol intake can lead to an increase in HDL-C levels [24]. Therefore, this relationship may be related to alcohol use. Although we found a nonlinear relationship between uric acid and HDL-C in hypertensive status, further basic experiments are needed to fully elucidate the specific biological mechanisms underlying this association.

There are some strengths in our study. First, we used adequate statistical analysis to examine the linear and nonlinear relationship between SUA and lipids. Second, considering that patients with eGFR <30 ml/min/1.73 m^2^ had abnormally high SUA levels, we excluded those with eGFR <30 ml/min/1.73 m^2^ in order to make our results more rigorous. Finally, compared with similar studies, we included a larger population and adjusted more confounding factors in the present study.

Some limitations should be noted. First, as a cross-sectional study, our results failed to provide causality regarding the relationship between SUA and lipids (including TG, LDL-C, and HDL-C). Second, we only quantitatively analyzed the relationship between SUA and lipids and did not collect information on the particle size, composition, function, and subclasses of TG, LDL-C, and HDL-C, so we could not conduct a more in-depth discussion. Third, we exclude study participants with eGFR <30 ml/min/1.73 m^2^. Hence, the study results may not be suitable for population with eGFR <30 ml/min/1.73 m^2^. Finally, we found a nonlinear relationship between SUA and HDL-C, but more basic research is needed to explain the mechanism of this finding.

## 5. Conclusion

In the present study, we found that SUA was positively associated with TG and LDL-C in hypertensive population with eGFR ≥30 ml/min/1.73 m^2^. The relationship between SUA and HDL-C was nonlinear. The negative correlation between SUA and HDL-C only existed when the SUA was less than 7.24 mg/dL in hypertensive population with eGFR ≥30 ml/min/1.73 m^2^. This finding suggests that clinicians must control the SUA level of hypertensive patients in the normal range. When the SUA level is within the normal range, SUA-lowering therapy can increase HDL-C levels in hypertensive patients, thereby obtaining greater clinical benefit.

## Figures and Tables

**Table 1:** Characteristics of study population.

Characteristics^*∗*^	Total	Tertiles of SUA (mg/dL)			*P* value
		T1 (<5.57)	T2 (5.57–8.39)	T3 (≥8.39)	
*N*	13,355	4,464	4,514	4,377	
Demographics					
Age (years)	63.77 ± 9.33	63.27 ± 8.79	64.10 ± 9.29	63.96 ± 9.86	<0.001
Male (%)	6301 (47.18)	1145 (25.65)	2226 (49.31)	2930 (66.94)	<0.001
Smoking (%)	3443 (25.79)	783 (17.54)	1235 (27.37)	1425 (32.56)	<0.001
Alcohol use (%)	2906 (21.77)	559 (12.53)	1025 (22.71)	1322 (30.21)	<0.001
Comorbidity (%)					
Stroke	923 (6.91)	303 (6.79)	307 (6.80)	313 (7.15)	0.747
CHD	689 (5.16)	193 (4.32)	232 (5.14)	264 (6.03)	0.004
Diabetes	2451 (18.35)	742 (16.62)	821 (18.19)	888 (20.29)	<0.001
Medication use (%)					
Antihypertensive drugs	8617 (64.54)	2757 (61.77)	2907 (64.41)	2953 (67.48)	<0.001
Lipid-lowering drugs	478 (3.58)	170 (3.81)	146 (3.23)	162 (3.70)	0.298
Glucose-lowering drugs	694 (5.20)	251 (5.62)	216 (4.79)	227 (5.19)	0.202
Physical examination					
BMI (kg/m^2^)	23.64 ± 3.75	23.18 ± 3.53	23.61 ± 4.07	24.12 ± 3.55	<0.001
SBP (mmHg)	148.41 ± 18.30	149.88 ± 17.52	148.52 ± 19.05	146.81 ± 18.15	<0.001
DBP (mmHg)	89.00 ± 11.61	88.53 ± 10.42	89.06 ± 13.14	89.41 ± 11.07	0.002
Biomarkers					
TC (mmol/L)	5.16 ± 1.11	5.09 ± 1.07	5.16 ± 1.10	5.22 ± 1.16	<0.001
TG (mmol/L)	1.80 ± 1.25	1.60 ± 1.00	1.78 ± 1.26	2.02 ± 1.43	<0.001
LDL-C (mmol/L)	2.98 ± 0.81	2.93 ± 0.79	2.99 ± 0.81	3.03 ± 0.84	<0.001
HDL-C (mmol/L)	1.57 ± 0.42	1.61 ± 0.43	1.56 ± 0.42	1.53 ± 0.42	<0.001
Hcy (*μ*mol/L)	17.70 ± 10.74	15.47 ± 8.30	17.66 ± 10.33	20.02 ± 12.69	<0.001
FBG (mmol/L)	6.18 ± 1.59	6.15 ± 1.73	6.15 ± 1.54	6.23 ± 1.48	0.023
AST (U/L)	26.74 ± 15.39	24.68 ± 9.57	26.79 ± 20.29	28.79 ± 14.03	<0.001
ALT (U/L)	20.47 ± 16.49	17.80 ± 10.86	20.57 ± 20.17	23.10 ± 16.62	<0.001
eGFR (ml/min/1.73 m^2^)	89.33 ± 18.16	97.11 ± 14.21	89.82 ± 16.58	80.91 ± 19.59	<0.001

Abbreviations: SUA, serum uric acid; CHD, coronary heart disease; BMI, body mass index; SBP, systolic blood pressure; DBP, diastolic blood pressure; TC, total cholesterol; TG, triglyceride; LDL-C, low-density lipoprotein cholesterol; HDL-C, high-density lipoprotein cholesterol; Hcy, homocysteine; FBG, fasting blood glucose; AST, aspartate aminotransferases; ALT, alanine transaminase; eGFR, estimated glomerular filtration rate. ^*∗*^Data are presented as number (%) or mean ± standard deviation.

**Table 2:** Association between SUA and lipids.

Variables	Crude model		Model 1		Model 2	
	*β* ^a^ (95% CI)	*P* value	*β* ^a^ (95% CI)	*P* value	*β* ^a^ (95% CI)	*P* value
*TG (mmol/L)*						
SUA (per 1 mg/dL change)	0.11 (0.10, 0.12)	<0.001	0.16 (0.15, 0.18)	<0.001	0.15 (0.14, 0.16)	<0.001
Tertiles						
T1 (<5.57)	Reference		Reference		Reference	
T2 (5.57–8.39)	0.18 (0.13, 0.23)	<0.001	0.31 (0.26, 0.36)	<0.001	0.26 (0.21, 0.31)	<0.001
T3 (≥8.39)	0.42 (0.37, 0.47)	<0.001	0.63 (0.58, 0.68)	<0.001	0.54 (0.48, 0.60)	<0.001
*P* for trend	<0.001		<0.001		<0.001	

*LDL-C (mmol/L)*						
SUA (per 1 mg/dL change)	0.03 (0.02, 0.04)	<0.001	0.06 (0.06, 0.07)	<0.001	0.06 (0.05, 0.07)	<0.001
Tertiles						
T1 (<5.57)	Reference		Reference		Reference	
T2 (5.57–8.39)	0.06 (0.03, 0.10)	<0.001	0.15 (0.12, 0.18)	<0.001	0.13 (0.10, 0.16)	<0.001
T3 (≥8.39)	0.10 (0.07, 0.14)	<0.001	0.25 (0.21, 0.28)	<0.001	0.22 (0.18, 0.25)	<0.001
*P* for trend	<0.001		<0.001		<0.001	

*HDL-C (mmol/L)*						
SUA (per 1 mg/dL change)	−0.02 (−0.03, −0.02)	<0.001	−0.02 (−0.02, −0.01)	<0.001	−0.01 (−0.01, 0.01)	0.083
Tertiles						
T1 (<5.57)	Reference		Reference		Reference	
T2 (5.57–8.39)	−0.05 (−0.07, −0.04)	<0.001	−0.05 (−0.06, −0.03)	<0.001	−0.02 (−0.04, −0.01)	0.021
T3 (≥8.39)	−0.09 (−0.11, −0.07)	<0.001	−0.07 (−0.09, −0.05)	<0.001	−0.02 (−0.04, 0.01)	0.107
*P* for trend	<0.001		<0.001		0.104	

Abbreviations: CI, confidence interval; SUA, serum uric acid; TG, triglyceride; LDL-C, low-density lipoprotein cholesterol; HDL-C, high-density lipoprotein cholesterol. Crude model: unadjusted; model 1: adjusted for age, sex; model 2: adjusted for age, sex, smoking, alcohol use, stroke, diabetes, antihypertensive drugs, lipid-lowering drugs, glucose-lowering drugs, BMI, SBP, DBP, Hcy, FBG and eGFR. ^a^Indicated effect sizes *(β)* were combined by Rubin's rule.

**Table 3:** The results of the two-piecewise linear model.

Outcome	*N*	HDL-C	
		*β* (95% CI)	*P* value
Fitting model by standard linear regression		−0.01 (−0.01, 0.01)	0.083
Fitting model by two-piecewise linear regression			
Inflection point of SUA (per 1 mg/dL change)		7.24	
<7.24 mg/dL	7,987	−0.02 (−0.02, −0.01)	<0.001
≥7.24 mg/dL	5,368	0.01 (−0.01, 0.02)	0.099
*P* for log likelihood ratio test		0.001	

Adjusted for age, sex, smoking, alcohol use, stroke, diabetes, antihypertensive drugs, lipid-lowering drugs, glucose-lowering drugs, BMI, SBP, DBP, Hcy, FBG, and eGFR.

## Data Availability

The datasets generated and analyzed during the current study are not publicly available because this study is still ongoing and the follow-up is not finished, but are available from the corresponding author on reasonable request.

## Abbreviations

SUA: Serum uric acid
TC: Total cholesterol
TG: Triglycerides
HDL-C: High-density lipoprotein cholesterol
LDL-C: Low-density lipoprotein cholesterol
eGFR: Estimated glomerular filtration rate
CHD: Coronary heart disease
FBG: Fasting blood glucose
Hcy: Homocysteine
SBP: Systolic blood pressure
DBP: Diastolic blood pressure
AST: Aspartate aminotransferases
ALT: Alanine transaminase
CI: Confidence interval.

## Supplementary Materials

Supplementary Materials Table S1: characteristics of study population. Table S2: results of subgroup analysis and interaction test (TG). Table S3: results of subgroup analysis and interaction test (LDL-C). Table S4: results of subgroup analysis and interaction test (HDL-C).
