# Supplementary material for: Is There a Nonlinear Relationship between Serum Uric Acid and Lipids in a Hypertensive Population with eGFR ≥30 ml/min/1.73 m2? Findings from the China Hypertension Registry Study
Source: Int J Endocrinol. 2020 Sep 18;2020:9725979. doi: 10.1155/2020/9725979 (PMC7519986; doi:10.1155/2020/9725979)
Supplement: Supplementary Materials — Table S1: characteristics of study population. Table S2: results of subgroup analysis and interaction test (TG). Table S3: results of subgroup analysis and interaction test (LDL-C). Table S4: results of subgroup analysis and interaction test (HDL-C). [file 9725979.f1.docx]

| Table S1. Characteristics of Study Population | | | | |
| --- | --- | --- | --- | --- |
| SUA, mg/dL* | Total | <7.24 | ≥7.24 | *P* value |
| N | 13,355 | 7,987 | 5,368 |  |
| Demographics |  |  |  |  |
| Age, years | 63.77 ± 9.33 | 63.63 ± 9.02 | 63.98 ± 9.76 | 0.033 |
| Male, % | 6301 (47.18) | 2822 (35.33) | 3479 (64.81) | <0.001 |
| Smoking, % | 3443 (25.79) | 1724 (21.59) | 1719 (32.03) | <0.001 |
| Alcohol use, % | 2906 (21.77) | 1317 (16.50) | 1589 (29.61) | <0.001 |
| Comorbidity, % |  |  |  |  |
| Stroke | 923 (6.91) | 550 (6.89) | 373 (6.95) | 0.889 |
| CHD | 689 (5.16) | 369 (4.62) | 320 (5.96) | <0.001 |
| Diabetes | 2451 (18.35) | 1386 (17.35) | 1065 (19.84) | <0.001 |
| Medication use, % |  |  |  |  |
| Antihypertensive drugs | 8617 (64.54) | 5021 (62.88) | 3596 (67.00) | <0.001 |
| Lipid-lowering drugs | 478 (3.58) | 284 (3.56) | 194 (3.61) | 0.859 |
| Glucose-lowering drugs | 694 (5.20) | 412 (5.16) | 282 (5.25) | 0.808 |
| Physical examination |  |  |  |  |
| BMI, kg/m^2^ | 23.64 ± 3.75 | 23.35 ± 3.83 | 24.06 ± 3.58 | <0.001 |
| SBP, mmHg | 148.41 ± 18.30 | 149.28 ± 18.33 | 147.13 ± 18.18 | <0.001 |
| DBP, mmHg | 89.00 ± 11.61 | 88.76 ± 12.01 | 89.36 ± 10.99 | 0.003 |
| Biomarkers |  |  |  |  |
| TC, mmol/L | 5.16 ± 1.11 | 5.12 ± 1.08 | 5.22 ± 1.15 | <0.001 |
| TG, mmol/L | 1.80 ± 1.25 | 1.66 ± 1.07 | 2.00 ± 1.46 | <0.001 |
| LDL-C, mmol/L | 2.98 ± 0.81 | 2.95 ± 0.79 | 3.03 ± 0.83 | <0.001 |
| HDL-C, mmol/L | 1.57 ± 0.42 | 1.59 ± 0.42 | 1.53 ± 0.42 | <0.001 |
| Hcy, μmol/L | 17.70 ± 10.74 | 16.41 ± 9.40 | 19.62 ± 12.22 | <0.001 |
| FBG, mmol/L | 6.18 ± 1.59 | 6.16 ± 1.68 | 6.20 ± 1.44 | <0.001 |
| AST, U/L | 26.74 ± 15.39 | 25.62 ± 16.53 | 28.41 ± 13.37 | <0.001 |
| ALT, U/L | 20.47 ± 16.49 | 19.02 ± 16.67 | 22.64 ± 15.98 | <0.001 |
| eGFR, ml/min/1.73m^2^ | 89.33 ± 18.16 | 94.18 ± 15.50 | 82.12 ± 19.38 | <0.001 |

Abbreviations: SUA, serum uric acid; CHD, coronary heart disease; BMI, body mass index; SBP, systolic blood pressure; DBP, diastolic blood pressure; TC, total cholesterol; TG, triglyceride; LDL-C, low density lipoprotein cholesterol; HDL-C, high density lipoprotein cholesterol; Hcy, homocysteine; FBG, fasting blood glucose; AST, aspartate aminotransferases; ALT, alanine transaminase; eGFR, estimated glomerular filtration rate.

*Data are presented as number (%) or mean ± standard deviation.

| **Table S2 Results of subgroup analysis and interaction test (TG)** | | | |
| --- | --- | --- | --- |
| Subgroup | N | Adjusted β (95%CI) | *P* for interaction |
| **TG, mmol/L** |  |  |  |
| Sex |  |  | 0.218 |
| male | 6,301 | 0.14 (0.12, 0.16) |  |
| female | 7,054 | 0.16 (0.14, 0.18) |  |
| Age, years |  |  | <0.001 |
| <65 | 6,623 | 0.20 (0.18, 0.22) |  |
| ≥65 | 6,732 | 0.11 (0.09, 0.13) |  |
| BMI, kg/m^2^ |  |  | <0.001 |
| <24 | 7,468 | 0.12 (0.10, 0.14) |  |
| ≥24 | 5,882 | 0.19 (0.17, 0.21) |  |
| Stroke |  |  | 0.300 |
| No | 12,432 | 0.15 (0.14, 0.17) |  |
| Yes | 923 | 0.13 (0.08, 0.17) |  |
| CHD |  |  | 0.349 |
| No | 12,666 | 0.15 (0.14, 0.16) |  |
| Yes | 689 | 0.13 (0.08, 0.18) |  |
| Diabetes |  |  | <0.001 |
| No | 11,980 | 0.14 (0.13, 0.15) |  |
| Yes | 1,375 | 0.23 (0.19, 0.26) |  |
| eGFR, ml/min/1.73m^2^ |  |  | <0.001 |
| <90 | 5,710 | 0.10 (0.08, 0.12) |  |
| ≥90 | 7,645 | 0.18 (0.17, 0.20) |  |
| Smoking |  |  | 0.970 |
| No | 9,909 | 0.15 (0.13, 0.17) |  |
| Yes | 3,443 | 0.15 (0.13, 0.17) |  |
| Alcohol use |  |  | 0.133 |
| No | 10,445 | 0.14 (0.13, 0.16) |  |
| Yes | 2,906 | 0.17 (0.14, 0.19) |  |

Adjusted for age, sex, smoking, alcohol use, stroke, diabetes, antihypertensive drugs, lipid-lowering drugs, glucose-lowering drugs, BMI, SBP, DBP, Hcy, FBG and eGFR, if not be stratified.

| **Table S3 Results of subgroup analysis and interaction test (LDL-C)** | | | |
| --- | --- | --- | --- |
| Subgroup | N | Adjusted β (95%CI) | *P* for interaction |
| **LDL-C, mmol/L** | |  |  |
| Sex |  |  | 0.136 |
| male | 6,301 | 0.05 (0.04, 0.07) |  |
| female | 7,054 | 0.07 (0.05, 0.08) |  |
| Age, years |  |  | 0.631 |
| <65 | 6,623 | 0.06 (0.05, 0.07) |  |
| ≥65 | 6,732 | 0.06 (0.05, 0.07) |  |
| BMI, kg/m^2^ |  |  | 0.061 |
| <24 | 7,468 | 0.05 (0.04, 0.07) |  |
| ≥24 | 5,882 | 0.07 (0.06, 0.08) |  |
| Stroke |  |  | 0.556 |
| No | 12,432 | 0.06 (0.05, 0.07) |  |
| Yes | 923 | 0.07 (0.04, 0.10) |  |
| CHD |  |  | 0.240 |
| No | 12,666 | 0.06 (0.05, 0.07) |  |
| Yes | 689 | 0.04 (0.01, 0.07) |  |
| Diabetes |  |  | 0.206 |
| No | 11,980 | 0.06 (0.05, 0.07) |  |
| Yes | 1,375 | 0.07 (0.05, 0.10) |  |
| eGFR, ml/min/1.73m^2^ |  |  | 0.246 |
| <90 | 5,710 | 0.05 (0.04, 0.06) |  |
| ≥90 | 7,645 | 0.06 (0.05, 0.07) |  |
| Smoking |  |  | 0.046 |
| No | 9,909 | 0.06 (0.05, 0.07) |  |
| Yes | 3,443 | 0.05 (0.03, 0.06) |  |
| Alcohol use |  |  | 0.336 |
| No | 10,445 | 0.06 (0.05, 0.07) |  |
| Yes | 2,906 | 0.05 (0.04, 0.07) |  |

Adjusted for age, sex, smoking, alcohol use, stroke, diabetes, antihypertensive drugs, lipid-lowering drugs, glucose-lowering drugs, BMI, SBP, DBP, Hcy, FBG and eGFR, if not be stratified.

| **Table S4 Results of subgroup analysis and interaction test (HDL-C)** | | | |
| --- | --- | --- | --- |
| Subgroup | N | Adjusted β (95%CI) | *P* for interaction |
| **HDL-C, mmol/L** | |  |  |
| Sex |  |  | 0.568 |
| male | 6,301 | -0.01 (-0.01, 0.01) | |
| female | 7,054 | -0.01 (-0.01, 0.01) | |
| Age, years |  |  | <0.001 |
| <65 | 6,623 | -0.01 (-0.02, -0.01) |  |
| ≥65 | 6,732 | 0.01 (-0.01, 0.01) |  |
| BMI, kg/m^2^ |  |  | 0.114 |
| <24 | 7,468 | -0.01 (-0.01, 0.01) |  |
| ≥24 | 5,882 | -0.01 (-0.02, -0.01) |  |
| Stroke |  |  | 0.453 |
| No | 12,432 | -0.01 (-0.01, -0.01) |  |
| Yes | 923 | 0.01 (-0.01, 0.02) |  |
| CHD |  |  | 0.209 |
| No | 12,666 | -0.01 (-0.01, -0.01) |  |
| Yes | 689 | 0.01 (-0.01, 0.02) |  |
| Diabetes |  |  | 0.453 |
| No | 11,980 | -0.01 (-0.01, -0.01) |  |
| Yes | 1,375 | -0.01 (-0.02, 0.01) |  |
| eGFR, ml/min/1.73m^2^ |  |  | 0.002 |
| <90 | 5,710 | -0.01 (-0.01, 0.01) |  |
| ≥90 | 7,645 | -0.01 (-0.02, -0.01) |  |
| Smoking |  |  | 0.311 |
| No | 9,909 | -0.01 (-0.01, -0.01) |  |
| Yes | 3,443 | -0.01 (-0.01, 0.01) |  |
| Alcohol use |  |  | 0.166 |
| No | 10,445 | -0.01 (-0.01, -0.01) |  |
| Yes | 2,906 | 0.01 (-0.01, 0.01) |  |

Adjusted for age, sex, smoking, alcohol use, stroke, diabetes, antihypertensive drugs, lipid-lowering drugs, glucose-lowering drugs, BMI, SBP, DBP, Hcy, FBG and eGFR, if not be stratified.
